# Chromosomal Inversions in Chromosome U of *Drosophila subobscura*: A Story from Population Studies to Molecular Level

**DOI:** 10.3390/insects16060586

**Published:** 2025-06-01

**Authors:** Mercè Merayo, Kenia M. Delgado, David Salguero, Dorcas J. Orengo

**Affiliations:** 1 Faculty of Computer Science, Multimedia and Telecommunications Universitat Oberta de Catalunya, Rambla del Poblenou, 156, 08018 Barcelona, Spain; mmerayo@uoc.edu (M.M.); kenia98@uoc.edu (K.M.D.); 2 Departament de Genètica, Microbiologia i Estadística, Universitat de Barcelona, 08028 Barcelona, Spain; dsalguero@ub.edu; 3 Institut de Recerca de la Biodiversitat (IRBio), Universitat de Barcelona, 08028 Barcelona, Spain

**Keywords:** *Drosophila subobscura*, chromosomal inversions, fold-back element

## Abstract

*Drosophila subobscura* is a Palearctic species that colonized the west coast of South and North America in the last quarter of the 20th century. This species stands out for its large chromosomal inversion polymorphism that affects its five long chromosomes. Studies of natural populations revealed that the inversion polymorphism has an adaptive character and while the arrangement U_st_ was classified as adapted to cold, other arrangements, such as U_1+2+6_ and U_1+8__+2_, were considered warm adapted. Characterization of the inversion breakpoints will allow a first approach to the genes included in the inversions and to find candidates to be affected by selection. In this work, we take advantage of the existence of a reference genomic sequence carrying the U_1+2_ arrangement to locate the breakpoints of the U_6_ and U_8_ inversions, mapping paired-end Illumina reads from two homokaryotypic strains to U_1+2+6_ and U_1+8__+2_, respectively. To date, most of the characterized inversion breakpoints in *D. subobscura* have been generated by non-homologous end-joining. In contrast, the U_6_ and U_8_ inversions seem to have originated by transposons, and, at the distal breakpoint of inversion U_8_, we describe a new fold-back-like element characteristic of the suboscura species group that we have named *Ziga-Zaga*.

## Simple Summary

*Drosophila subobscura* is a Palearctic species that colonized the west coast of both Americas in the last quarter of the 20th century. This species stands out for its large chromosomal inversion polymorphism that affects its five long chromosomes. Studies of natural populations in these three geographic areas revealed that the inversion polymorphism has an adaptive character. However, there is no information on which genes may be involved in the different climatic adaptations. Characterization of the inversion breakpoints will allow a first approach to the genes included in the inversions and to find candidates to be affected by selection. In this work, we take advantage of the existence of a reference genomic sequence that carries the U_1+2_ arrangement to localize the breakpoints of inversions U_6_ and U_8_. Our results suggest that the origin of both inversions would have been mediated by transposons and, in the case of U_8_, we found a new fold-back-type element characteristic of the subobscura species.

## 1. Introduction

Chromosomal inversions represent an important structural variation both within species and in speciation processes. They consist of two breaks in a chromosome that leave a fragment free, and, when repaired, the ends are joined so that this fragment is inserted in the opposite direction within the chromosome. Inversions were inferred more than 100 years ago by Sturtevant in *Drosophila* comparing genetic maps built from recombination rates [1] and their existence was later confirmed by cytological studies of polytene chromosomes. Polytene chromosomes are giant chromosomes typical of some Diptera tissues that are made up of numerous DNA replicas that remain together without the cells dividing. In addition, the two homologous chromosomes also remain paired. This means that, when there is a heterozygote for a chromosomal inversion, these polytene chromosomes form inversion loops that are detected cytologically [2]. For this reason, chromosomal inversions have been studied mainly in Diptera and very especially in different species of *Drosophila* [3,4].

**Figure 1:**
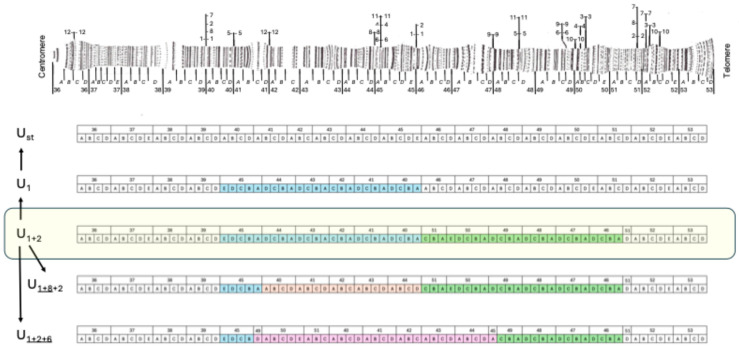
Kunze-Mühl and Müller [8] cytological map of chromosome U_st_ of *D. subobscura* and a schematic representation of four successive inversions on this chromosome. The ancestral character of the U_1+2_ arrangement is indicated by yellow shading and, on the left, the sequential relationship among the arrangements is indicated.

In Europe, the study of inversions has been carried out extensively in *Drosophila subobscura* Collin and its twin species *D. madeirensis* Monclús (endemic to Madeira) and *D. guanche* Monclús (endemic to the Canary Islands). The karyotype of this group of species consists of five long acrocentric chromosomes named A (=X), J, U, E and O, and a point-shaped chromosome named dot, and these three species differ from each other by a series of fixed inversions. The most common arrangement of each chromosome in northern European populations was named the standard arrangement and is therefore not necessarily the ancestral arrangement [5]. For example, the ancestral arrangement of chromosome U of *D. subobscura* (B Muller element) corresponds to the U_1+2_ arrangement, which is the only one present in *D. madeirensis* and *D. guanche* and one of the polymorphic variants in *D. subobscura* [6]. This chromosome shows a rich structural polymorphism by inversions that, in most of the Mediterranean natural populations analyzed, consists of five arrangements: U_st_, U_1_, U_1+2_, U_1+8__+2_ and U_1+2+6_ [5,7]. These arrangements differ by two tandem inversions (U_1_ and U_2_, which cytologically share a breakpoint) and other overlapping inversions (Figure 1).

Whereas the endemic species of this group are monomorphic, *D. subobscura* is a species with a large chromosomal polymorphism by inversions affecting its five long acrocentric chromosomes. This species was originally from the Palearctic region with a wide distribution from southern Scandinavia to northern Africa and from the Caucasus to the Macaronesian islands, where its chromosomal inversion polymorphism has been widely studied [5]. The latitudinal clinal variation of this polymorphism in Europe suggested an adaptive origin that would be confirmed when, in the last quarter of the 20th century, the species colonized the Pacific coast of both Chile and North America and rapidly replicated these clines [9]. This adaptive character is also supported by the fact that the variation in chromosomal polymorphism over time correlates with temperature changes in the same direction as the latitudinal clines [7,10,11].

Inversions may affect the fitness of individuals mainly in two ways. On the one hand, inversions could directly break a gene or affect its expression by separating the gene from its regulatory region. On the other hand, since inversions block recombination in heterokaryotypes [12], they can hold together specific combinations of coadapted alleles of the genes included in an inversion [13]. Although several studies point to an adaptive origin of the inversion polymorphism in *D. subobscura*, the genes responsible for the adaptive character of the inversions and their clinal variation are still unknown. A first step in this direction must be the molecular characterization of the breakpoints that delimit the inversions. In the last decade, when the genomic sequence of this species was not yet available, several studies aimed at the molecular characterization of the inversion’s breakpoints of chromosomes O, E and A of *D. subobscura*, obtaining probes from a chromosome walking on the genome of *D. pseudoobscura* and performing in situ hybridization [14,15,16,17,18,19]. Once the genome of *D. guanche* was available [20], a chromosomal walking in this genome sequence was used to characterize the fixed inversions (in chromosomes A, J, E and O) between *D. guanche* and *D. subobscura* [21]. Finally, Karageorgiou and collaborators obtained a genomic sequence for *D. subobscura* from a homokaryotipic line for the arrangements A_st_, J_st_, U_st_ E_st_ and O_3+4_. These authors performed a whole genome synteny analysis of this genome with that of *D. guanche* and obtained the breakpoints of all the fixed inversions between these two species identified before by more classical methods [21] and for the inversions U_1_ and U_2_ (that are polymorphic in *D. subobscura*) [22]. Their results for U_1_ and U_2_ inversions show clear evidence of an origin by staggered breaks that gave rise to duplicated sequences in opposite orientations of 689 and 1007 bp in length, respectively. Furthermore, their results show that these two tandem inversions, which cytologically share a breakpoint, are separated by about 31 kb [22].

The distribution of inversions along chromosomes is not completely random and several of them have some shared breakpoint, at least at the cytological level [8]. Furthermore, many of the molecularly characterized breakpoints have revealed the presence of duplicated sequences in opposite orientation flanking the inverted region [23]. Depending on the nature of these duplicated sequences, two main mechanisms have been proposed for the origin of the inversions. When the duplicated sequences are repetitive sequences, as for example with transposable elements, inversions may have arisen from non-allelic homologous recombination (NAHR) between these repeated sequences. On the other hand, when the duplicated sequences are non-repetitive fragments, and they are duplicated only in one of the two arrangements, the duplicated sequence could be the consequence of a non-homologous end-joining (NHEJ) of two staggered breaks [23]. Both mechanisms seem to act to varying degrees in genome evolution depending on the species. However, in the case of *D. subobscura*, the molecular characterization of several breakpoints has only detected clear examples of NHEJ [21,22]. In addition, other less frequent and/or more complex mechanisms may occur at the origin of some inversions. In this sense, it has recently been seen that two inversions of *D. subobscura* have required the intervention of two homologous chromosomes at their origin [24,25].

In the last decade, with the growth in next-generation sequencing, several methodologies have been developed to detect structural variants from paired-end sequences [26,27,28]. In short, clusters of read pairs that do not map to the reference genome in the expected orientation and distance to each other are indicative of the existence of structural variants (SVs). In the present work, we have used this strategy to characterize the breakpoints of two inversions of chromosome U of *D. subobscura* (the U_6_ and U_8_ inversions). We chose these two inversions to apply this bioinformatic strategy because on chromosome U only inversions U_1_ and U_2_ had been previously characterized. On the other hand, both U_6_ and U_8_ are the most common inversions that originate in a U_1+2_ arrangement in the Mediterranean area, and we were able to obtain homokaryotypic strains for these inversions. Furthermore, we can take advantage of the fact that the reference genome of *D. subobscura* [29] carries the U_1+2_ arrangement to find the breakpoints of these two inversions (U_6_ and U_8_) by directly comparing the reference genomic sequence with those of the U_1+2+6_ and U_1+8__+2_ arrangements. In the case of the U_1+8__+2_ arrangement, there are two more very interesting characteristics: (1) it seems to be adapted to warm climates [30,31]; and (2) one of its breakpoints is shared with inversions U_1_ and U_2_ [8]. Our objective is, therefore, the molecular characterization of the breakpoints of these two inversions. This work will contribute to knowledge of the origin of the inversions and, although it does not focus directly on the adaptation of inversions, it may be a first step to understanding their selective maintenance in nature.

## 2. Materials and Methods

### 2.1. Drosophila Strains

The homokaryotypic strains of *D. subobscura* used in this study were obtained through over 13 generations of sibmating from isofemale lines established upon collection in Observatori Fabra (Barcelona, Catalonia, Spain), as reported in [15]. Specifically, the sequenced strains were OF28 and OF58, which are homokaryotypic for the U_1+2+6_ and U_1+8__+2_ chromosomal arrangements, respectively. In addition, strain OF74, which is homokaryotypic for the U_1+2_ arrangement, was also used to validate the bioinformatic results using polymerase chain reactions (PCRs).

### 2.2. DNA Extraction and Sequencing

For each strain, genomic DNA was extracted from 20 adult individuals using the Puregen Cell kit B (Qiagen, Hilden, Germany). Two and four micrograms of genomic DNA for strains OF28 and OF58, respectively, were sent to CNAG (Barcelona, Spain) for further sample preparation and sequencing. Libraries for paired-end Illumina reads, separated by ~300 bp inserts, were indexed for multiplex sequencing on the same sequencing lane of a HiSeq 2500 flowcell (Illumina Inc., San Diego, CA, USA) and sequenced in PE mode and 2 × 101 nt read length. A total of 41,333,785 and 67,671,844 reads were obtained, respectively.

### 2.3. Bioinformatics of Sequencing Data

**Figure 2:**
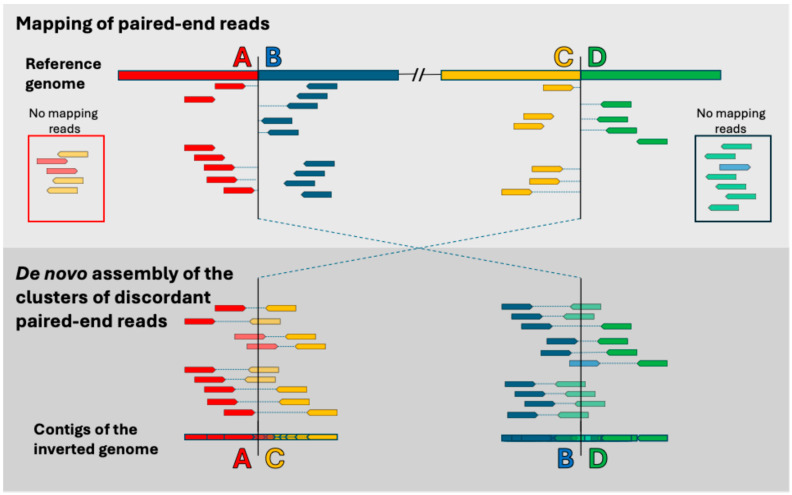
Schematic representation of the paired-end reads mapping around the breakpoints of a chromosomal inversion. Reads from each paired-end are indicated by two colored arrows joined by a dashed line (pairs are red-orange or blue-green, depending on the region where they map). (**Top**) Reads mapping positions on the reference sequence in the AB and CD regions. Around breakpoints in the reference sequence, reads map into clusters of discordant pairs (without the expected orientation and with a mapped distance much larger than the average insert size). In addition, reads spanning breakpoints are not mapped. In the boxes are reads that do not map to any part of the reference genome (in lighter color). (**Bottom**) The inferred positions on the inverted haplotype (regions AC and BD) of the clusters of discordant paired-end reads and the contigs obtained by their de novo assembly.

To map the two breakpoints of an inversion (i.e., regions AB and CD in the reference genome and regions AC and BD in the inverted genome, Figure 2), we used a strategy already applied to *D. melanogaster* [26,27], which consists of detecting clusters of discordant paired-end reads when mapping to the reference genome. It is expected that the reassembling de novo of these discordant reads will recover the inverted points AC and BD.

**Figure 3:**
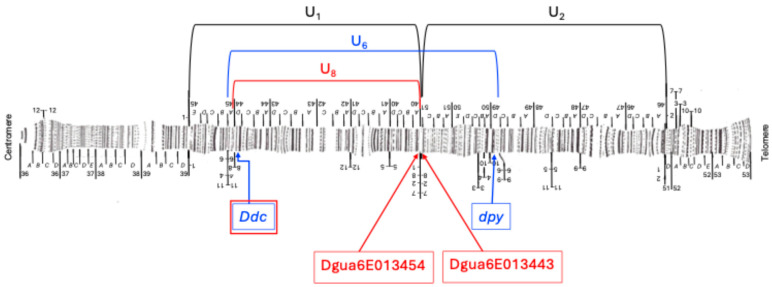
Location of genetic markers near the U_6_ (indicated in blue boxes) and U_8_ (indicated in red boxes) inversion breakpoints mapped in previous studies on Kunze-Mühl and Müller’s cytological map [8] of the *D. subobscura* U chromosome. Note that the U chromosome map has been modified to show the U_1+2_ arrangement. Also, note that the *Ddc* gene was used as a marker for both inversions.

The bioinformatics workflow followed is presented in Supplementary Materials Figures S1 and S2, and the scripts used are available on https://github.com/mercemerayo/Dsubobscura-inversions; accessed on 30 March 2025. This workflow consists of five main steps: (1) quality control, (2) mapping to a reference genome; (3) detecting of discordant paired-end reads; (4) de novo assembling of the discordant reads; (5) verification of the contigs. The first step of quality control and preprocessing of the reads was performed with fastp [32]. Then, the processed reads were mapped to a “hologenome” of reference consisting of the genomes of *D. subobscura* (UCBerk Dsub 1.0)*, Saccaromyces cerevisae* and other possible microbial symbionts or pathogens following the list used by Kapun and collaborators [33], where other possible members of the *Drosophila* microbiome [34,35] have been added (Supplementary Materials Table S1). Mapping to a hologenome allows for the rapid removal of reads corresponding to microorganisms that it includes, avoiding them interfering with the correct mapping of the rest of the reads. The mapping was performed using the software BWA-MEM2 (version 2.2.1) [36] and the duplicated reads were marked using Samtools version 1.19.2 [37]. The mapping of the U chromosome was then analyzed using the BreakDancer program (version 1.4.5) [38]. This software detects paired-end reads that map in a discordant way and returns an output consisting of a list of coordinates where clusters of such discordant reads occur. This list was filtered for possible inversions expanding through the coordinates of known molecular markers near the cytological breakpoints of the inversion of interest. Table 1 shows the information of the molecular markers used to search for the inversion breakpoints and Figure 3 shows their location on the chromosome U used as the genome of reference.

Once the coordinates of the putative inversions were determined, paired-end reads mapping around 5 kb on each side were extracted with Samtools (version 1.19.2) and de novo reassembled using SPAdes (version 4.0.0) [40]. We used two types of read pairs for the assembly. The first type included pairs where both reads were mapped to the target regions. The second included pairs in which one read mapped within 5 kb of either putative breakpoint, while the other read remained unmapped. These unmapped reads are of great interest, as they are expected to cross the breakpoint (Figure 2).

Finally, the recovered contigs were aligned to the *D. subobscura* reference genome [29] using the Basic Local Alignment Search Tool (BLAST) on the webpage of NCBI [41] to assign their relative location in the reference genome and infer their location in the inverted genome (Supplementary Materials Figures S3 and S4).

### 2.4. PCR Validation and Sanger Sequencing

For each inversion analyzed (U_6_ and U_8_), we identified putative contigs that crossed the breakpoints in the inverted chromosome, or two concatenated contigs that were expected to align in the corresponding manner. These contigs were used to design amplification primers for the regions AC and BD, using the Primer-BLAST facility on the NCBI webpage that uses Primer3 (version 2.5.0). Before choosing a primer pair, the presence of each oligo was also tested for also in the reference genome to be sure that a correct combination of the four primers would also obtain amplification for regions AB and CD in a strain with the U_1+2_ arrangement (Supplementary Materials Tables S2 and S3).

A total of eight PCR amplifications were performed for each inversion: AB, CD, AC and BD, both for the OF74 strain carrying the U_1+2_ arrangement and for the strain with the additional inversion (OF28 or OF58, depending on the tested inversion U_6_ or U_8_, respectively). In the case of the OF74 strain, only two PCRs should be positive (AB and CD) and with an amplification band of known size. In the case of the other strain, only the combinations of primers AC and BD should give an amplification band and, in some cases, their sizes are unknown.

For the BD fragment of the U_1+8__+2_ arrangement, the bioinformatics approach generated two unconnected contigs. As a result, a primer walking sequencing was initiated to resolve these fragments further. Since the original BD primers generated a faint secondary amplification band (Supplementary Materials Figure S5) and some of the sequencing primers did not work properly, a pair of internal PCR primers (Bi and Di, Supplementary Materials Table S2) were designed to obtain a nested reamplification PCR in an attempt to achieve greater specificity of DNA amplification. In all cases, amplicons were purified with Exo-SapIT (Amersham Biosciences, Piscataway, NJ, USA) prior to their sequencing with the ABI PRISM version 3.2 cycle sequencing kit (Applied Biosystems, Foster City, CA, USA). Sequencing products were separated using an ABI PRISM 3730 sequencer (Perkin-Elmer, Norwalk, CT, USA). Sequences were assembled using the DNASTAR package [42].

The sequences newly obtained for regions AC and BD in the arrangement U_1+8__+2_ have been deposited in the European Nucleotide Archive (ENA) under project number PRJEB88772.

## 3. Results

### 3.1. Inversion U_6_

For the OF28 strain (U_1+2+6_), the BreakDancer software (version 1.4.5) returned a list of 166 possible inversions in the chromosome U, 92 of which had a score higher than 99. Among these, an inversion at coordinates 7,714,784 and 16,614,075 stands out, which is consistent with the molecular markers we had for the inversion U_6_ (Table 1). In addition, this inversion was one of those supported by a higher number of reads (83). The reassembling of the reads that mapped around these coordinates in the reference genome gave a total of 55 contigs with only four of them having a length greater than 5 kb. Contig_1 with a length of 11,791 bp crosses the BD region, while contig_4 with 6421 bp crosses the AC breakpoint in the arrangement U_1+2+6_. It should be noted that, when performing BLAST with the obtained contigs, the breakpoints that are recovered are slightly shifted with respect to those indicated by BreakDancer. Thus, the proximal breakpoint would be between positions 7,714,148 and 7,714,441 of chromosome U of the reference genome instead of position 7,714,784 predicted by BreakDancer. On the other hand, the distal breakpoint would be between positions 16,613,602 and 16,613,622 instead of the position 16,614,075. In both cases the recovered points are approximately 400 bp before those predicted (Supplementary Materials Figure S3).

**Figure 4:**
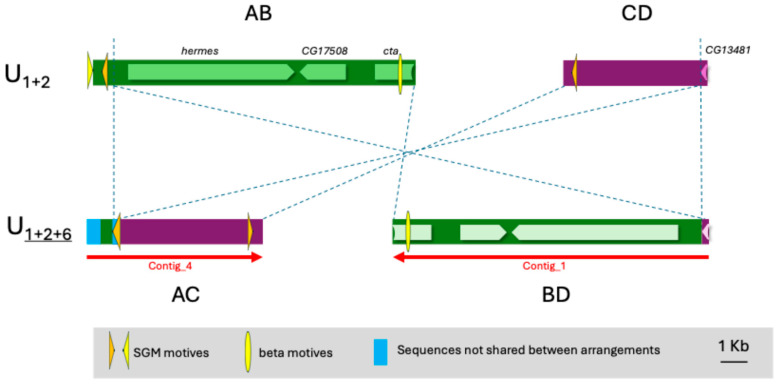
Schematic representation of inversion U_6_ breakpoint regions in chromosomal arrangements U_1+2_ and U_1+2+6_ and their functional annotation. Red arrows represent the recovered contigs. The names of the genes correspond to the orthologous genes in *D. melanogaster*. Genes on the BD regions are indicated with a fainter color because they are only inferred from the overall sequence similarity. The orange and yellow triangles show the starting and ending sequences of the SGM motif, respectively.

Both breakpoints are in intergenic regions and no clear duplications were found in either the original or inverted arrangements (Figure 4). The proximal breakpoint is near the LOC117902107 gene, and the distal breakpoint is close to the 3′ end of the LOC117900774 gene. These genes are orthologous to the *hermes* and CG13481 genes in *D. melanogaster,* respectively.

At the proximal breakpoint, there is a partial SGM element [43] and Contig_4 has an initial region (1–489) with a sequence that does not match to any of the original AB or CD regions.

The PCR performed with the primers designed to validate the inversion produced several bands, indicating the possible presence of repetitive sequences in these regions. It is important to keep in mind that, although both Contig_1 and Contig_4 cross the breakpoints in the inverted arrangement, they extend mainly on the internal part of the inversion (regions B and C, respectively). Regions A and D only are covered by 465 and 271 nucleotides, respectively, which makes it difficult to design suitable primers for performing the PCR validation of the breakpoints.

### 3.2. Inversion U_8_

**Figure 5:**
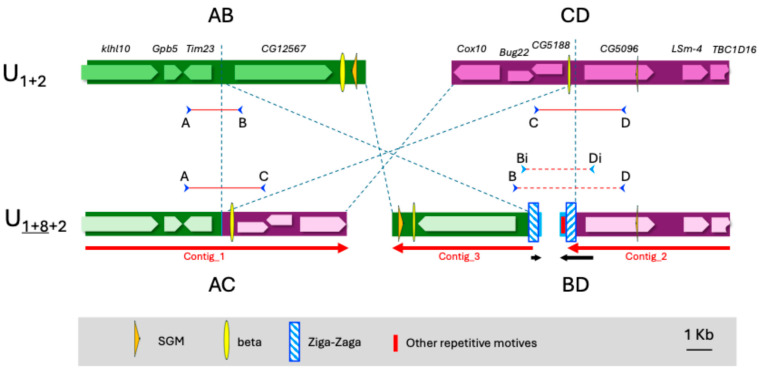
Schematic representation of inversion U_8_ breakpoint regions in chromosomal arrangements U_1+2_ and U_1+8__+2_. Red arrows represent the recovered contigs from Illumina paired-end reads. The black arrows show the sequences obtained by Sanger sequencing. Genes on the inverted arrangement are indicated with a fainter color because they are only inferred from overall sequence similarity. The orange triangles show the starting sequences of the SGM motif. Blue arrowheads show the location of the PCR primers used to validate the breakpoints.

In the case of the OF58 strain (U_1+8__+2_), the BreakDancer software gave a list of 205 possible inversions in the chromosome U, 90 of which had a score higher than 99. Among these, an inversion at coordinates 7,772,887 and 14,722,269 stands out, which is consistent with the molecular markers we had for the inversion U_8_ (Table 1). In addition, this inversion was one of those supported by a higher number of reads (48). The reassembling of the reads that mapped around these coordinates in the reference genome yielded a total of four contigs. The contig_1 with a length of 10,248 bp crosses the AC breakpoint in the inverted arrangement (U_1+8__+2_) since its first 5334 nucleotides align directly in region A (from 7,767,475 to 7,772,833) of the chromosome U of the reference genome, while its last part aligns in the inverted direction in region C (from 14,716,861 to 14,721,766) of the same chromosome (Figure 5). Contig_2 with 6368 bp aligns only to region B and Contig_3 with 5481 bp aligns only to region D. On the other hand, the BLAST analysis with the Contig_4 of only 314 bp does not return any resemblance with the reference genome. Figure 5 shows that the three longest contigs cover practically the entire AB and CD regions of the reference genome, leaving only 141 and 12 nucleotides uncovered, respectively. In these uncovered regions (7,772,834–7,772,973 for the proximal point and 14,721,767–14,721,777 for the distal point) must be located the breakpoints in the original arrangement. It should be noted that the proximal point fits well with the BreakDancer prediction, while the distal point was predicted to be ~500 bp more distal. Another point that arises from our results is that the distal breakpoint is ~100 kb more distal than the proximal breakpoint of inversion U_2_ (Table 1). Thus, inversions U_1_ and U_2_ would overlap inversion U_8,_ and the arrangement should be named U_1+2+8_.

Both breakpoints in the U_1+2_ arrangement are in intergenic regions, but near genes. The proximal point is flanked by the gene *Tim23* and the orthologous to *CG12567* of *D. melanogaster*. The distal point falls between two predicted genes orthologous to *D. melanogaster,* CG5188 and CG5096, respectively.

In the inverted genome, the AC region has been assembled continuously, but the sequence in the BD region is discontinuous and would probably have incorporated new sequences, such as those recovered by Contig_4. In fact, the last ~320 nucleotides of contig_2 and the first ~130 of Contig_3 do not match the original AB or CD regions and have some similarities to multiple sequences in the genome.

Four oligonucleotides, one in each region (A, B, C and D), were designed to test the correct assignment of contigs (Supplementary Materials Table S2 and Figure 5). As expected, the combinations of primers AB and CD only gave an amplification band when using DNA from the strain OF74 that has the original arrangement U_1+2_. On the other hand, also as expected, the combinations of primers AC and BD only gave an amplification band when using DNA from the strain OF58 that carries the arrangement U_1+8__+2_ (Supplementary Materials Figure S5). The recovered bands AB, CD and AC have the sizes expected from the known sequences. For its part, the band obtained for the region BD, in the inverted arrangement, shows a size of approximately 4 kb, indicating that a sequence of ~1.3 kb has been inserted between Contig_2 and Contig_3.

Sequencing of the BD fragment was initiated by primer walking, but several problems were encountered in completing this sequence. To begin with, the PCR band obtained was much weaker than that of the other fragments tested (Supplementary Materials Figure S5). Attempts to improve band intensity by adjusting the annealing temperature and designing reamplification primers (Figure 5) were unsuccessful. In addition, some sequencing primers did not work, and others only allowed obtaining about 300 bp and then the sequence quality suddenly dropped. Therefore, only 436 bp could be added to the sequence of the beginning of Contig_3 (end of subregion B of the BD region) and 269 bp at the end of Contig_2 (beginning of subregion D of the BD region). The BLAST analysis of the complete sequence obtained revealed that subregions B and D of the inverted genome contain repeated fragments in inverted sense. Thus, a 371 bp fragment at the end of subregion B (136 nucleotides at the beginning of Contig_3 and 235 added by Sanger sequencing) and 371 bp at the beginning of subregion D (323 bp at the end of Contig_2 plus 48 added by Sanger sequencing) show a 97% identity in the reversed direction. In addition, part of these inverted sequences gave a BLAST alignment (92% identity) with the sequence close to one of the breakpoints for the fixed inversion Af3 between chromosomes X of *D. subobscura* and *D. guanche* that could not be completely sequenced [21].

Given the impossibility of continuing sequencing towards the interior of the BD region, we changed our strategy and attempted to sequence from the interior of the BD fragment outwards. Assuming that Contig_4 (which did not align anywhere in the original arrangement) had to be found between contigs 3 and 2 in the inverted arrangement, we designed amplification primers that would allow us to check this hypothesis and decide in which direction Contig_4 is located. The PCR results would seem to indicate that Contig_4 is located between the two long contigs in the reverse direction, but the sizes of the bands obtained do not fit our expectations and none of the amplification primers were able to yield a clear sequence.

We also investigated whether the sequences around the breakpoints contained any of the repetitive sequences described in *D. subobscura*, i.e., SGM (Miller et al. 2000 [43]) and α, β or δ motifs [15]. Two fragments of SGM of ~200 and ~60 bp were found in the regions B and D, respectively, and also two small fragments of the β motif of ~130 and ~170 bp were found in the regions B and C, respectively (Figure 5).

### 3.3. Description of “Ziga-Zaga”, a Fold-Back Element in the Subobscura Group

The difficulties that we found in completing the BD sequence led us to find a sequence of 371 bp long that is repeated in an inverted orientation next to the BD breakpoint. This structure, along with the difficulties in obtaining a strong PCR band in this region, suggests that this could be a fold-back element. BLAST analysis of this 371 bp sequence in NCBI revealed that it has no homology to any sequence in the general database. When searched within specific genomes, it is not found in either the *Drosophila melanogaster* or *D. pseudoobscura* genomes, and its occurrence appears to be restricted to the *D. subobscura* group. In this group of species, some TEs and satellite sequences have already been described as SGM [43] and sat290 [44], but the sequence found here has no similarities to them. Therefore, we named this new FB element *Ziga-Zaga*, where each unit of 371 bp will be *Ziga* or *Zaga*, and when they are found together, form the *Ziga-Zaga* element. This sequence of 371 bp seems to correspond to a partial *Ziga* repeat since when using the whole 472 bp of the sequence that we know occurs at the end of the B subregion, the BLAST matches include this additional 101 bp that are not found at the beginning of the subregion D. On the other hand, BLAST of the initial 592 bp of the D region produces two types of matches: those already expected for its final fragment of 371 bp (222–592) and another collection of matches for the first 221 nucleotides.

When searching for very similar sequences (megablast) with alignments of more than 50 bp, it is found that the modular 371 bp sequence (*Ziga* or *Zaga*) occurs 98 times in the *D. subobscura* genome distributed across all chromosomes. Many of these repeated sequences are paired in the genome and, when this is the case, most (30 pairs) are in inverted orientation and separated by a variable length, ranging from 130 to 5854 bp (Supplementary Materials Table S4).

The genomes of *D. madeirensis* and *D. guanche* also have this *Ziga-Zaga* element on all their chromosomes, indicating that its appearance predates speciation in this group. The number of copies detected by BLAST in the *D. madeirensis* genome is 68 (lower than that of *D. subobscura*), and it has many copies on the O chromosome (Supplementary Materials Table S4) and especially in its last 35 kb where five partial copies (from 124 to 371 bp) are found in tandem orientation and separated by variable lengths no longer than 2.2 kb. In the case of *D. guanche*, the BLASTN was performed on the CNAG website (https://denovo.cnag.cat/dguanche; accessed on 12 April 2025) since the results obtained through NCBI do not correspond to chromosomes but to scaffolds. After filtering for alignments longer than 50 bp, we found 121 matches (Supplementary Materials Table S4). This number is higher than that obtained for *D. subobscura*, but many of these matches (34) correspond to alignments that do not exceed 100 bp. In addition, even considering these short alignments, the fold-back structure was only found in 13 instances. Another observation is that at the extremes of the chromosomes, a higher number of these repeats seem to be concentrated but in the same orientation. This is the case of the chromosome A telomere with three tandem partial copies.

Next to the *Zaga* fragment found in region D of the U_1+8__+2_ arrangement is a fragment of 221 nucleotides that is also a repetitive sequence. As pointed out above, when doing BLAST for the whole 592 nucleotide sequence at the beginning of subregion D, two collections of different matches are obtained. For the first 221, a total of 56 matches were obtained in the *D. subboscura* genome. All of them are close to a match of the *Ziga* or *Zaga* sequences, but their relative position and orientation is very variable. The same pattern is observed in *D. madeirensis* that has 32 matches for this sequence. It highlights that the telomere of chromosome O seems to be enriched with this sequence alternating with the *Zaga* motifs. As this sequence of 221 bp is always close to a *Ziga* motive, we named it *company* (companion in Catalan).

## 4. Discussion

Several studies have revealed that the rich chromosomal inversion polymorphism in *D. subobscura* is modeled by natural selection [9] and particularly mediated by thermal adaptation [7,10,11,29,45,46,47,48,49]. However, it is not known which genes are being directly selected along with each inversion. A first step in this direction must be the molecular localization of the inversion breakpoints. In this study, we have focused on the characterization of two U chromosome inversions (U_6_ and U_8_). The first does not seem to be particularly adapted to certain climatic conditions since its frequency of occurrence in natural populations is low, but the U_8_ inversion is considered to be well adapted to warm climates [29,47,50].

### 4.1. Limitations of the Methods Used to Molecularly Characterize Inversions and Their Breakpoints

Before the genome sequence of *D. subobscura* was available, several studies were carried out using laborious methodologies of chromosome walking and in situ hybridization in order to locate probes that crossed each of the breakpoints of some of its most common chromosomal inversions [14,15,16,17,18,19]. Once a complete genomic sequence was available, the breakpoints of three more inversions could be found by a synteny analysis comparing two complete genomes, either from related species [22] or from individuals carrying other chromosomal arrangements [25]. The present study adds information in this regard for two inversions of the U chromosome (U_6_ and U_8_), exploring the utility of a new approach to the problem, mapping paired-read sequences from individuals carrying an inversion with respect to the reference genome.

This approach has been used by Cridland and Thornton in *D. melanogaster* to identify diverse structural variants [26]. Specifically, these authors could differentiate between three kinds of SV: indels, Class 1 events (tandem duplications or translocations) and Class 2 events (inversions or duplications with a change in orientation). Regarding Class 2 events, they state that most of those detected could be artifacts of the sequencing process, reinforcing the importance of having multiple read pairs supporting each event. Lately, Corbett-Detig et al. have presented a method that uses the mapping of multiple samples to detect inversions and their breakpoints in population samples [27]. This approach allowed them to confirm the breakpoints of three previously characterized inversions and localize those of four more. Nevertheless, one of the breakpoints of both inversions *In(2L)t* and *In(3R)P* could not be recovered and it could be due to the presence of repetitive elements adjacent to these breakpoints [51,52]. The only drawback of the method of Corbett-Detig et al. is that inversions present in only one individual will be missed.

We have returned to the original method of searching for clusters of discordant reads by mapping sequences from a single strain using BreakDancer, which is one of several programs that have been developed for this purpose [28]. This methodology has proven to be robust enough to recover the breakpoints of a fixed inversion between *D. subobscura* and *D. guanche* in chromosome J [53]. In this case, the sequences obtained for the de novo assembly of the discordant reads of *D. subobscura* around the predicted breakpoints in *D. guanche* perfectly match the sequences obtained by the traditional method of chromosome walking and in situ hybridization [21]. In the present study, we applied this approach to find the breakpoints of two long polymorphic inversions of the U chromosome and this has allowed the molecular characterization of one of them (U_8_). However, our results for the U_6_ inversion are inconclusive, as the recovered contigs only partially cross the breakpoints of the inverted genome. The recovery of these “chimeric” contigs (with BLAST alignments of different fragments to one or another region of the reference genome) seems to confirm the location of the breakpoints, but the shortness of the sequence in one of the two regions did not allow us to design suitable primers for PCR validation. Furthermore, the assembling of the reads yielded a high number of small contigs, which may be indicative of the presence of repetitive sequences in the inverted genome. In fact, BLAST of some of these contigs, such as Contig_5 of 3251 bp showed a high similarity of its sequence to multiple sites not only on chromosome U but also on the rest of the chromosomes. This makes it unfeasible to fully conclude the molecular characterization of these breakpoints in this way.

Regarding the U_8_ chromosomal inversion, the proposed strategy yielded better results, allowing us to molecularly characterize its breakpoints. The selected reads from the mapping to the reference U chromosome could be assembled into three long contigs covering the two original regions AB and CD (Supplementary Materials Figure S4), and a short contig of 314 bp with a sequence that does not exist in the ancestral arrangement. In this case, the proximal breakpoint recovered by our sequence fits well with the point predicted by BreakDancer. On the other hand, the distal point is displaced by about 500 bp, which is a distance greater than the expected 95% confidence interval, which would be twice the standard deviation of the insert size [54], which for this sample is 213. The region around the proximal breakpoint in the inverted arrangement (AC) was determined directly from one of the recovered contigs. However, to complete the sequence of the distal breakpoint (BD), PCR amplification and Sanger sequencing of the central region were required. Several problems were encountered in completing this sequence. To begin with, the PCR band obtained was much weaker than that of the other fragments tested (Supplementary Materials Figure S5). Our efforts to obtain a more intense band were futile, both by varying the annealing temperature and by designing reamplification primers. In addition, some sequencing primers did not work; and others only allowed us to obtain a fragment of about 300 bp and then the sequence quality suddenly dropped. The attempt to sequence from Contig 4, which appears to be in the central region of the missing sequence, also yielded no results. The origin of the problem seems to be in the existence of a region duplicated in the inverted orientation that could be hindering both correct amplification and obtaining suitable sequencing primers. This region has similarities to another present near a fixed inversion between *D. subobscura* and *D. guanche* and which also prevented its complete characterization. These observations seem to point to the presence of a fold-back element that could be involved in the origin of the inversion (see below).

Thus, we can conclude that, although the method used allows the recovery of inversion breakpoints, it does not allow the complete molecular characterization of those involving complex repetitive sequences. In these cases, the complete characterization will require the use of long-read sequencing technologies to circumvent the major problems found in repetitive regions (the assembly of short reads or finding good primers to proceed with Sanger sequencing). In the case of the BD region of inversion U_8_, the PCR results indicate that a stretch of ~1.3 kb is missing between the two contigs of the Illumina assembly. A long read should allow us to connect these two contigs and decipher if there is any duplicated sequence with respect to the AC region that could clearly indicate the molecular origin of the inversion.

### 4.2. Origin of the Inversions U_6_ and U_8_

Two main mechanisms have been proposed as the origin of inversions: non-allelic homologous recombination (NAHR) and non-homologous end-joining (NHEJ).

In the case of NAHR, the inversion arises from recombination between sequences of the same chromatid that are duplicated in an inverted direction and flanking the fragment that undergoes inversion. Thus, both arrangements, the standard and the inverted, carry these flanking inverted sequences. Most of these inverted sequences correspond to transposable elements (TEs). In addition, TEs may generate chromosomal inversions through their transpositional activity, leaving TE inserts at one or both inversion breakpoints [55,56]. In *D. melanogaster*, several instances of the role of transposons have been identified [57,58], and also the origin of inversions in *D. buzzati* appears to be largely mediated by the action of transposons [59,60].

On the other hand, the NHEJ consists in the repair of two breaks in the same chromatid that may be blunt or staggered, and, in which case, will generate a duplication of a fragment of one or both original sequence breakpoints in the inverted arrangement in an inverted direction. This mechanism seems to be prevalent in the melanogaster species group [23] and in the subobscura species group [21]. In some cases, the mechanism of origin has had to be more complex, involving more than one chromatid, as has been inferred in *D. subobscura* for the E_9_ and O_7_ inversions [24,25].

In this work, we have analyzed two inversions of the U chromosome of *D. subobscura* at the molecular level. Although for the U_6_ inversion, the characterization has not been complete, it seems that there are no duplicated sequences in either the ancestral or the inverted arrangement. On the other hand, the presence of a partial SGM element [43] close to the breakpoint in Contig 4 along with a ~500 bp fragment whose sequence does not match any of the original AB or CD regions could indicate that the origin of this inversion is mediated by a transposable element. In the case of inversion U_8_, a new fold-back-like element has been characterized. The BD breakpoint region has not been completely sequenced, but the whole inverted repeat of *Ziga*-*Zaga* has been obtained and other repetitive sequences seem also to be present. It is interesting to note that the AC region includes an intermediate stretch of 34 nucleotides (5334–5368 in contig_1) that does not correspond to either of the two original subregions A or C (Supplementary Materials Figure S4). This sequence forms an almost perfect palindromic sequence with a high identity to the 21 first nucleotides of the *Ziga* unit (Supplementary Materials Figure S6). Since this stretch of sequence is very short (34 bp), its presence here could be a simple coincidence. However, the fact that it corresponds precisely to the outermost nucleotides of the *Ziga-Zaga* element along with their precise location right in the middle of the sequences where a break and repair has occurred suggests that it could be related to the origin of the inversion (and could be the footprint of the previous presence of a *Ziga-Zaga* element). Our conclusion is that the U_8_ inversion could have originated through the mediation of the *Ziga*-*Zaga* element. In cases where TEs are involved, it is almost impossible to know whether it was produced by NAHR or by the TE transposition process itself. Since the inversion event, the sequences have been able to evolve, preventing us from seeing the traces left by the process that generated it. On the other hand, since the complete sequence of the BD region is unknown, the possibility that the origin of the inversion was by NEHJ cannot be completely ruled out.

## 5. Conclusions

In this work, we present a bioinformatics pipeline that appears to be a reliable approach for obtaining the breakpoints of inversions in *D. subobscura*. However, sequences cannot be fully recovered when complex repetitive sequences are involved in the origin of the inversions.

To date, most of the inversions characterized at the molecular level in *D. subobscura* have originated from the repair of staggered end breaks generating inverted repeats in the new arrangement. The two inversions studied here show features consistent with a possible origin mediated by transposons, suggesting for the first time in this species that such a mechanism may play a role in inversion origination.

In fact, at the distal breakpoint of the U_8_ inversion, there is a folding element that prevented us from completing the sequence of this region. BLAST analysis of this sequence reveals that it is characteristic of the subobscura species group and confirms that this repetitive sequence is mostly present with a folding structure. Thus, we described a new fold-back-like element, which we have named *Ziga-Zaga*.

## Figures and Tables

**Table 1:** Cytological localization of the breakpoints of U_6_ and U_8_ inversions in *D. subobscura*, and cytological and genomic localization of molecular markers near these breakpoints.

Inversion	Marker	Band	Reference	BLAST
U_6_	Proximal BP.	45B/45A	[8]	¿?
	*Ddc*	44D	[39]	7,888,695–7,890,041
	*dpy*	49D	[20]	16,420,251–16,503,087
	Distal BP.	49D/49C	[8]	¿?
U_8_	Proximal BP.	45A/44D	[8]	¿?
	*Ddc*	44D	[8]	7,888,695–7,890,041
	Proximal BP. U_1_ (*Dgua6E013454)*	40A	[8,22]	14,589,270–14,593,607
	Distal BP. U_2_(*Dgua6E013443*)	51C	[8,22]	14,634,213–14,639,290
	Distal breakpoint	51C/40A	[8]	¿?

Note: ¿? Means unknowns location.

## Data Availability

The scripts used are available on https://github.com/mercemerayo/Dsubobscura-inversions (accessed on 30 March 2025). The sequences newly obtained for regions AC and BD in the arrangements U_1+2+6_ and U_1+8__+2_ will be deposited in the European Nucleotide Archive (ENA) under project number PRJEB88772.

## Supplementary Materials

The following supporting information can be downloaded at: https://www.mdpi.com/article/10.3390/insects16060586/s1, Figure S1: Workflow used to detect the inversion breakpoints.; Figure S2: Workflow used to reconstruct and validate the inversion breakpoints; Figure S3: Schematic representation of inversion U_6_ breakpoint regions; Figure S4: Schematic representation of inversion U_8_ breakpoint regions; Figure S5: Agarose gel with the PCR products; Figure S6: Alignment of the intermediate AC sequence of inversion U_8_ to the *Ziga-Zaga* element; Table S1: Microorganisms included in the hologenome of reference.; Table S2: PCR primers designed to amplify breakpoint regions of inversion U_8_; Table S3: Expected amplification bands; Table S4: Abundance of the *Ziga-Zaga* element in the genome of three species.
