# Supplementary material for: Chromosomal Inversions in Chromosome U of Drosophila subobscura: A Story from Population Studies to Molecular Level
Source: Insects. 2025 Jun 1;16(6):586. doi: 10.3390/insects16060586 (PMC12192754; doi:10.3390/insects16060586)
Supplement: Supplementary file 1 [file insects-16-00586-s001.zip › Supplementary Figure S1.pdf]

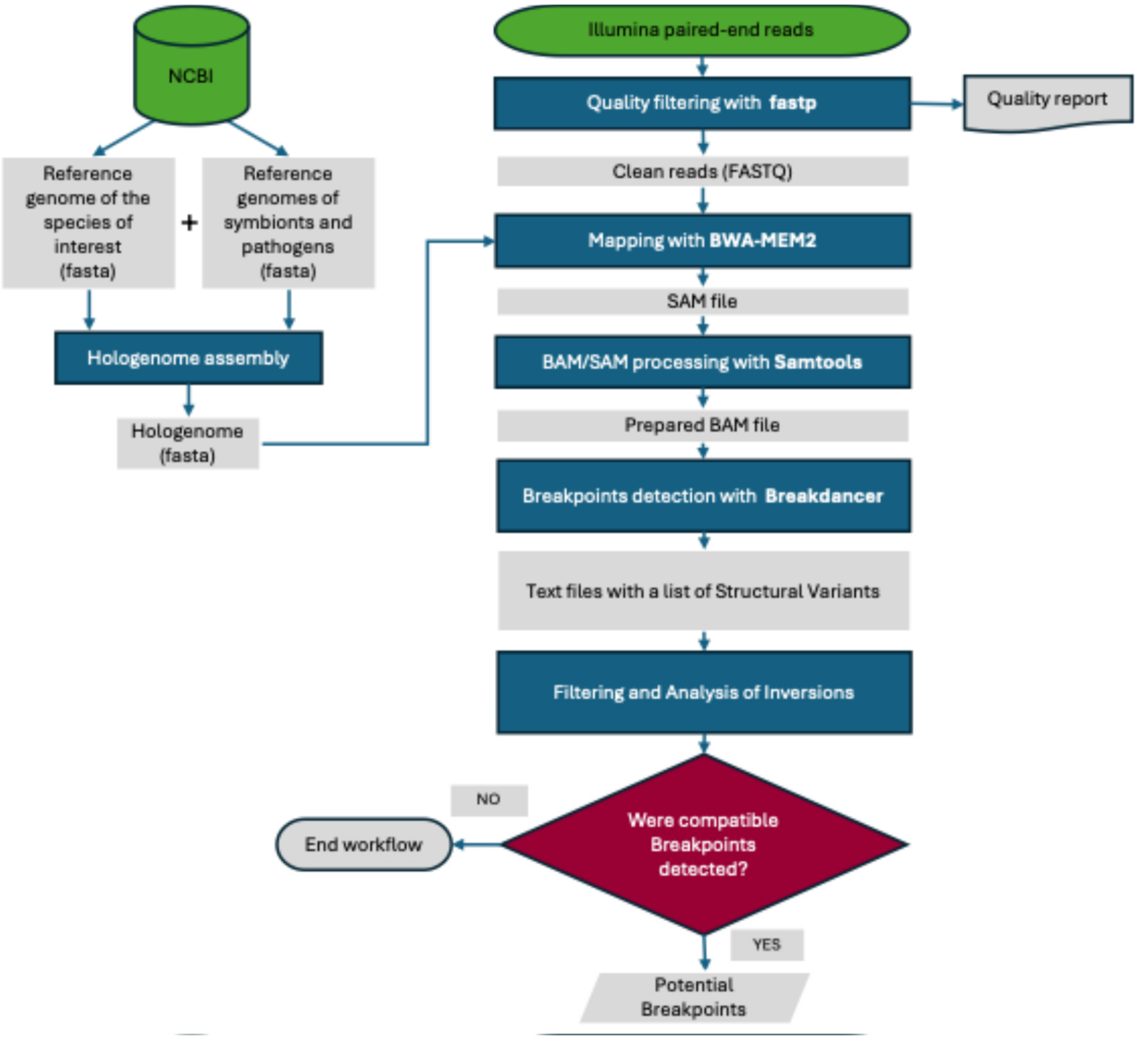

Supplementary Figure S1. Workflow used to detect the inversion breakpoints. Initial data are indicated in green; programs and bioinformatics processes are indicated in blue and intermediate data and final results are shown in grey.
