# Supplementary material for: Chromosomal Inversions in Chromosome U of Drosophila subobscura: A Story from Population Studies to Molecular Level
Source: Insects. 2025 Jun 1;16(6):586. doi: 10.3390/insects16060586 (PMC12192754; doi:10.3390/insects16060586)
Supplement: Supplementary file 1 [file insects-16-00586-s001.zip › Supplementary Figure S2.pdf]

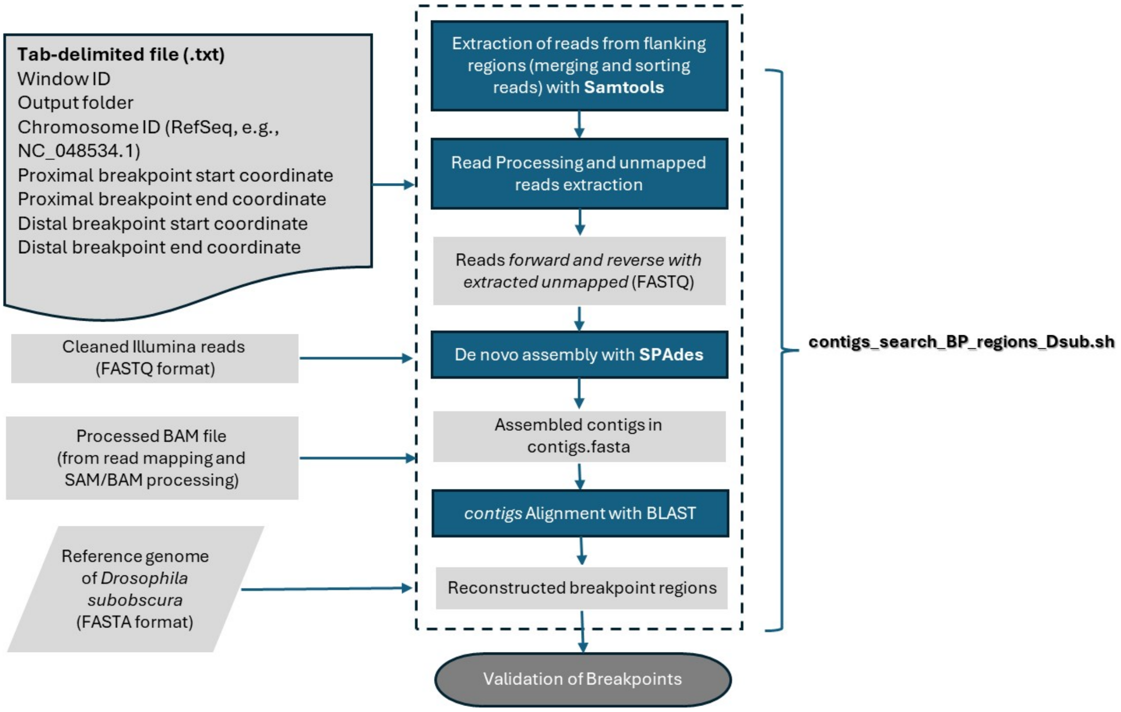

Supplementary Figure S2. Workflow used to reconstruct and validate the inversion breakpoints. Input data, shown in light-grey (outside the dashed area), include cleaned Illumina reads, BAM alignment files, reference genome, and a tab-delimited file with genomic data and output folders path. Bioinformatic tools and processing steps are indicated in blue within the dashed box. Output files, such as assembled contigs (contigs.fasta) and filtered BLAST alignments, are organized in dedicated directories. Final results, shown in dark grey, correspond to these outputs.
