# Supplementary material for: Chromosomal Inversions in Chromosome U of Drosophila subobscura: A Story from Population Studies to Molecular Level
Source: Insects. 2025 Jun 1;16(6):586. doi: 10.3390/insects16060586 (PMC12192754; doi:10.3390/insects16060586)
Supplement: Supplementary file 1 [file insects-16-00586-s001.zip › Supplementary Figure S4.pdf]

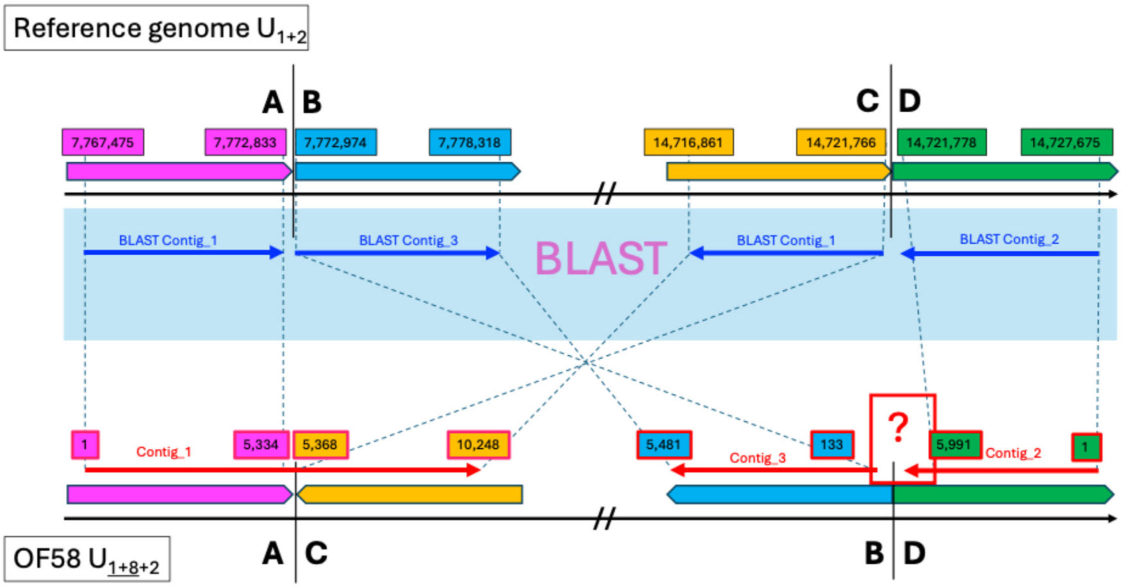

Supplementary Figure S4. Schematic representation of inversion U_8 breakpoint regions (not at scale) in chromosomal arrangements U_{1+2} and $U_{\underline{1+8}+2}$. Red arrows represent the recovered contigs while blue arrows show the BLAST alignments to the reference genome. The red box with a "?" indicates the sequence fragment inserted during the repair of the breaks. The numbers indicate the coordinates of the reference genome and the positions of the contigs corresponding to the ends of the BLAST-aligned fragments.
