# Supplementary material for: Chromosomal Inversions in Chromosome U of Drosophila subobscura: A Story from Population Studies to Molecular Level
Source: Insects. 2025 Jun 1;16(6):586. doi: 10.3390/insects16060586 (PMC12192754; doi:10.3390/insects16060586)
Supplement: Supplementary file 1 [file insects-16-00586-s001.zip › Supplementary Figure S5.pdf]

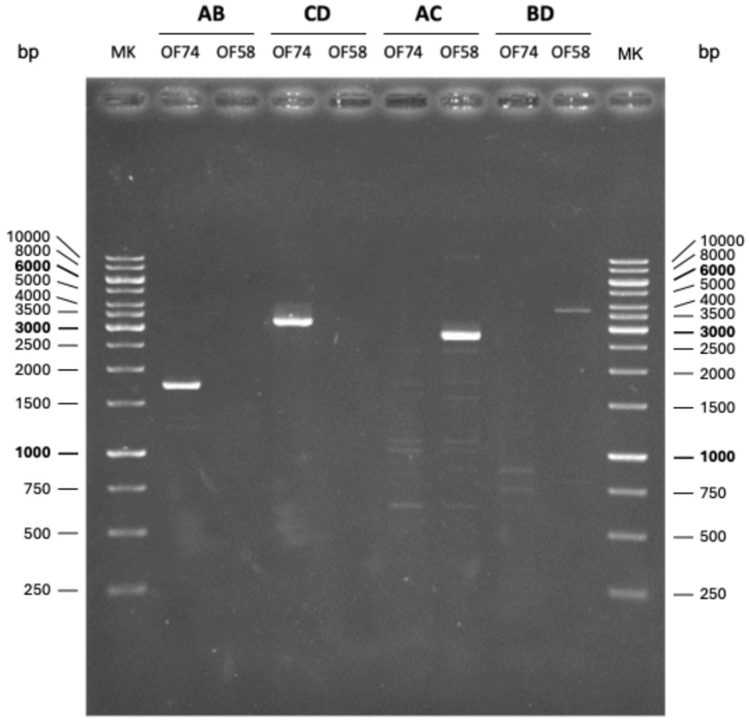

Supplementary Figure S5. Agarose gel with the PCR products obtained in the four combinations of primers AB, CD, AC and BD for strains OF74 (U_{1+2}) and OF58 (U_{1+8+2}). MK labels the molecular weight marker lanes with the band lengths indicated in number of base pairs (bp)at both sides of the gel.
