# Supplementary material for: Chromosomal Inversions in Chromosome U of Drosophila subobscura: A Story from Population Studies to Molecular Level
Source: Insects. 2025 Jun 1;16(6):586. doi: 10.3390/insects16060586 (PMC12192754; doi:10.3390/insects16060586)
Supplement: Supplementary file 1 [file insects-16-00586-s001.zip › Supplementary Figure S6.pdf]

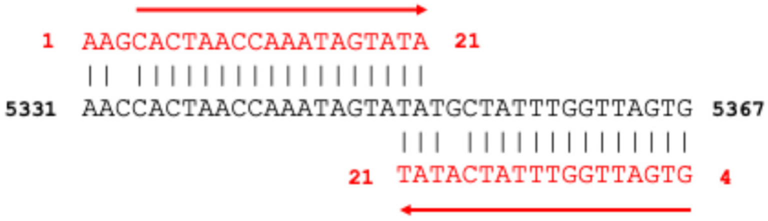

Supplementary Figure S6. Alignment of the intermediate AC sequence of inversion U_8 (in black) to the *Ziga-Zaga* element (in red). The red arrows show the direction of the *Ziga* unit.
