# Supplementary material for: Chromosomal Inversions in Chromosome U of Drosophila subobscura: A Story from Population Studies to Molecular Level
Source: Insects. 2025 Jun 1;16(6):586. doi: 10.3390/insects16060586 (PMC12192754; doi:10.3390/insects16060586)
Supplement: Supplementary file 1 [file insects-16-00586-s001.zip › Supplementary Table S1.pdf]

Supplementary Table S1. Microorganisms included in the hologenome of reference.

**Species name**	**RefSeq assembly accession**	
*Saccharomyces cerevisiae**	GCF 000146045.2	yeast
*Wolbachia pipientis**	GCF 007971685.1	bacteria
*Pseudomonas entomophila**	GCF 000026105.1	bacteria
*Commensalibacter intestini**	GCF 000231445.1	bacteria
*Acetobacter pomorum**	GCF 002738225.1	bacteria
*Gluconobacter morbifer**	GCF 000234355.1	bacteria
*Providencia burhodogranariea**	GCF 000314855.2	bacteria
*Providencia alcalifaciens**	GCF 002393505.1	bacteria
*Providencia rettgeri**	GCF 019048105.1	bacteria
*Enterococcus faecalis**	GCF 000393015.1	bacteria
Lactiplantibacillus plantarum	GCF 009913655.1	bacteria
Levilactobacillus brevis	GCF 900475625.1	bacteria
Hanseniaspora uvarum	GCA 037102615.1	yeast
Pichia kudriavzevii	GCF 003054445.1	yeast
Candida milleri = Maudiozyma humilis	GCA 037102785.1	yeast
Candida sake	GCA 003243815.1	yeast
Rhodotorula babjevae	GCA 920104245.2	yeast
Serratia liquefaciens	GCF 000422085.1	bacteria
Trigonopsis vinaria	GCA 030582095.1	yeast
Acetobacter aceti	GCF 000379545.1	bacteria
Leuconostoc mesenteroides	GCF 000014445.1	bacteria
Enterobacter cloacae	GCF 905331265.2	bacteria
Klebsiella oxytoca	GCF 900636985.1	bacteria
Erwinia carotovora = Pectobacterium carotovorum	GCF 013488025.1	bacteria
Zygosaccharomyces bailii	GCA 949129075.1	yeast

*microrganisms that have been used in an hologenome of *D. melanogaster* (Kapun et al 2016)
