# Supplementary material for: Chromosomal Inversions in Chromosome U of Drosophila subobscura: A Story from Population Studies to Molecular Level
Source: Insects. 2025 Jun 1;16(6):586. doi: 10.3390/insects16060586 (PMC12192754; doi:10.3390/insects16060586)
Supplement: Supplementary file 1 [file insects-16-00586-s001.zip › Supplementary Table S2.pdf]

Supplementary Table S2. PCR primers designed to amplify breakpoint regions of inversion U_8

name	sequence	length	Tm
A	CTCCGTAGGCCAGCTGAAAA	20	60.04
C	GGACGCATTTTGCCACCAAT	20	60.04
B	CCTGACTCTCCCGTCCAGTA	20	60.03
D	CCCAAGCCTGAAATTGCCAC	20	60.04
Bi	AAAGCGAAACGTCACAGTAGA	21	57.9
Di	AACAAACAAAACGTAGGTAAA	21	52.1
