# Supplementary material for: Chromosomal Inversions in Chromosome U of Drosophila subobscura: A Story from Population Studies to Molecular Level
Source: Insects. 2025 Jun 1;16(6):586. doi: 10.3390/insects16060586 (PMC12192754; doi:10.3390/insects16060586)
Supplement: Supplementary file 1 [file insects-16-00586-s001.zip › Supplementary Table S3.pdf]

Supplementary Table S3. Expected amplification bands

Region	forward primer	reverse primer	strain	arrangement	expected size
AC	A	C	OF_58	U_{1+8+2}	2.7 kb
	A	C	OF_74	U_{1+2}	no band
BD	B	D	OF_58	U_{1+8+2}	¿? =>2.7 kb
	B	D	OF_74	U_{1+2}	no band
AB	A	B	OF_58	U_{1+8+2}	no band
	A	B	OF_74	U_{1+2}	1.8 kb
CD	C	D	OF_58	U_{1+8+2}	no band
	C	D	OF_74	U_{1+2}	3.2 kb
