# Supplementary material for: Chromosomal Inversions in Chromosome U of Drosophila subobscura: A Story from Population Studies to Molecular Level
Source: Insects. 2025 Jun 1;16(6):586. doi: 10.3390/insects16060586 (PMC12192754; doi:10.3390/insects16060586)
Supplement: Supplementary file 1 [file insects-16-00586-s001.zip › Supplementary Table S4.pdf]

Supplementary Table S4. Abundance of the *Ziga-Zaga* element in the genome of three species.

*D. subboscura*

| chromosome | length | repeats | *Ziga-Zaga* | insert | tandem pairs |
|---|---|---|---|---|---|
| A | 24,182,865 | 32 | 9 | 1052 - 4024 | |
| J | 23,815,339 | 9 | 2 | 1155 - 1280 | |
| U | 25,941,769 | 15 | 5 | 888 -5854 | |
| E | 20,343,353 | 26 | 9 | 859 - 1835 | |
| O | 30,159,154 | 14 | 5 | 130 - 1832 | |
| dot | 1,505,893 | 2 | - | | |
| Total | 125,948,373 | 98 | 30 | | |

*D. madeirensis*

| chromosome | length | repeats | *Ziga-Zaga* | insert | tandem pairs |
|---|---|---|---|---|---|
| A | 24,006,601 | 15 | 3 | 630 - 992 | |
| J | 24,201,710 | 3 | 1 | 1688 | |
| U | 26,201,808 | 13 | 4 | 992 - 6862 | |
| E | 20,557,154 | 18 | 4 | 865 - 3028 | 1 |
| O | 30,799,563 | 17 | 1 | 877 | 7 (2 + 5) |
| dot | 1,488,072 | 2 | - | | |
| Total | 127,254,908 | 68 | 13 | | |

*D. guanche*

| chromosome | length | repeats | *Ziga-Zaga* | insert | tandem pairs |
|---|---|---|---|---|---|
| A | 22,902,232 | 45 | 6 | 178-1091 | 5 (2+3) |
| J | 23,034,698 | 12 | 1 | 4390 | 2 |
| U | 24,764,194 | 22 | 1 | 15 | 4 |
| E | 19,457,539 | 9 | 2 | 571- 840 | |
| O | 29,604,737 | 29 | 3 | 1 - 691 | |
| dot | 1,279,380 | 4 | - | | |
| Total | 121,042,780 | 121 | 13 | | |

Length, number of nucleotides in the assambled chromosomes; repeats, number of sequences with significant alignment to the 371 bp sequence; *Ziga-Zaga*, number of paired units in the correct orientation; insert, number of nucleotides between the *Ziga* and *Zaga* units; tandem pairs, number of pairs of units in the same orientation and separated by less than 5 Kb
