# Supplementary figures and images for: Chromosomal Inversions in Chromosome U of Drosophila subobscura: A Story from Population Studies to Molecular Level
Source: Insects. 2025 Jun 1;16(6):586. doi: 10.3390/insects16060586 (PMC12192754; doi:10.3390/insects16060586)

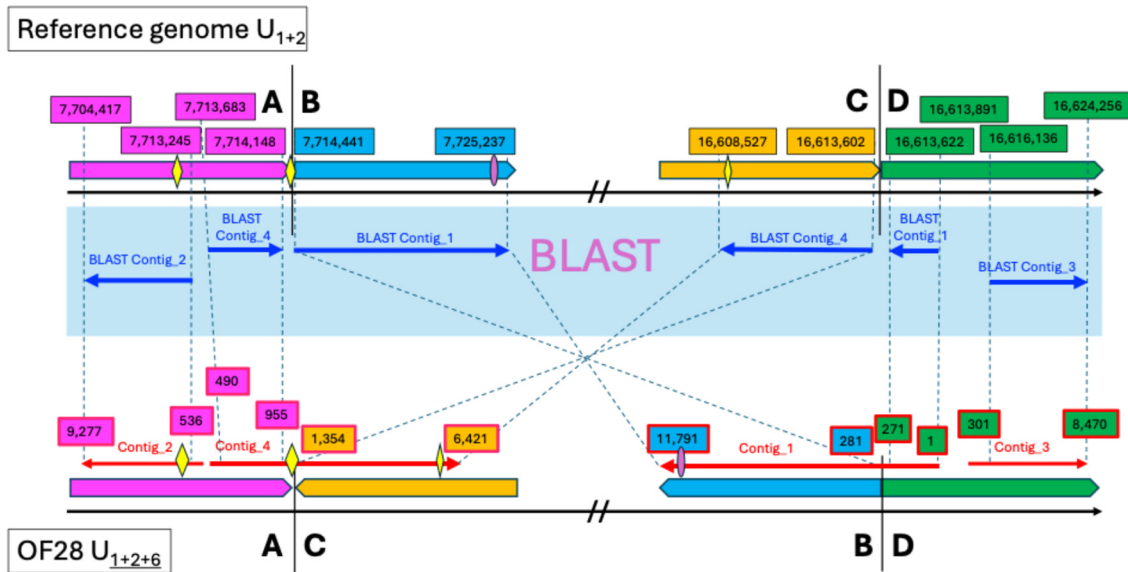

Supplement: Supplementary file 1 [file insects-16-00586-s001.zip › Supplementary Figure S3.pdf]
